# Adaptation of the Cyst Nematode *Globodera pallida* to the Colinear Potato Resistant QTLs *GpaV*_*vrn*_ and *GpaV*_*spl*_ Involved Distinct Genomic Regions and Absence of Cross‐Virulence

**DOI:** 10.1111/mec.70105

**Published:** 2025-09-13

**Authors:** Océane Lechevalier, Magali Esquibet, Mathieu Gautier, Rachel Fourdin, Eric Grenier, Sylvain Fournet, Josselin Montarry

**Affiliations:** ^1^ IGEPP, INRAE, Institut Agro Univ Rennes Le Rheu France; ^2^ CIRAD, INRAE, IRD, Institut Agro Montpellier CBGP, Univ Montpellier Montpellier France; ^3^ I2MC, UMR1297 INSERM Toulouse France

**Keywords:** adaptation, cross‐virulence, experimental evolution, genome scan, *Globodera pallida*, selection

## Abstract

The use of alternative methods to control cyst nematode populations has accelerated since the ban of chemical nematicides in Europe. The resistant QTL *GpaV*_*vrn*_, derived from the wild species *Solanum vernei* , is widely present in resistant European potato cultivars and provides strong protection against *Globodera pallida* populations although a risk of resistance breakdown has already been demonstrated in both experimental evolution studies and field populations. The wild relative *S. sparsipilum*, harbouring the resistant QTL *GpaV*_*spl*_, would be an interesting alternative source of resistance to control virulent *G. pallida* . The goal of the present study was to understand the genomics of adaptation of the nematode to these two colinear resistant QTLs. Starting with two natural populations, an experimental evolution approach allowed, after 10 generations on resistant potato genotypes, selecting independent nematode lineages adapted to each QTL. These virulent lineages were analysed through a combination of phenotyping and genome scans approaches. Phenotyping enabled the quantification of virulence levels and confirmed resistance breakdowns. Pool‐Seq whole genome sequencing followed by genome scan analyses identified genomic regions under selection, potentially involved in the adaptive mechanisms to each resistance factor. Candidate genes within these regions provided insights into the genetic basis of adaptation, revealing effectors known to suppress plant immunity. As genome scans highlighted distinct genomic regions for the adaptation to both resistant factors, we were able to predict and phenotypically confirm the absence of cross‐virulence between nematode lineages evolving on *GpaV*_*vrn*_ and *GpaV*_*spl*_. These findings have significant implications for the design of effective and sustainable resistance management strategies.

## 1 Introduction

In the last decade, agriculture in Europe has undergone a significant transformation under the influence of more stringent environmental regulation. The gradual withdrawal of many chemical pesticides, in particular all the most effective nematicides, has left producers with limited options for managing plant parasitic nematodes. In this new context, plant genetic resistance has emerged as a central pillar of sustainable crop protection strategies. Even though resistance can provide high levels of protection, it is not a perfect control option. One major limitation of this control option is the evolutionary potential of nematodes to overcome plant resistance. When pathogen populations display high evolutionary potential due to elevated genetic diversity, large population sizes and/or rapid reproductive cycles, they can quickly respond to the selective pressure imposed by resistant host plants (e.g., Montarry et al. 2021). Resistance breakdown that is, the selection of virulent individuals able to multiply on the resistant cultivar, can occur over just a few generations when pest populations have been subjected to selection pressure following repeated exposure to the same resistant factor (Castagnone‐Sereno et al. 2015; Fournet et al. 2013; Phillips and Blok 2008). By predicting the evolutionary trajectories of pathogen populations, it becomes possible to design resistance deployment strategies that are more sustainable and resilient over time.

The urgent need for anticipatory strategies is particularly evident for the potato cyst nematode *Globodera pallida*, as resistance breakdowns have already been reported in natural populations. Indeed, virulent populations (i.e., populations able to multiply on resistant cultivars) have been reported in Germany and in The Netherlands (Mwangi et al. 2019; Niere et al. 2014), suggesting resistance breakdown is no longer theoretical but a process already underway. This problem is compounded by the fact that most of the resistant European potato cultivars currently deployed rely on the same source of resistance, the quantitative locus *GpaV*_*vrn*_ derived from the wild species *Solanum vernei* . Experimental evolutionary approaches have also confirmed the ability of cyst nematode populations to adapt to plant resistance (Fournet et al. 2013; Kwon et al. 2024). Under controlled selection pressure over several generations, *G. pallida* populations evolved towards virulence to the resistance QTL *GpaV*_*vrn*_ (Fournet et al. 2013; Lechevalier et al. 2025). Conversely, the resistant QTL *GpaV*_*spl*_, derived from *Solanum sparsipilum*, has not yet been deployed in potato cultivars, so we have limited information on the ability of nematode populations to overcome it. Moreover, as these two QTLs are located in a collinear position on the potato chromosome V (Caromel et al. 2005; Rouppe van der Voort et al. 2000), investigating potential cross‐virulence between these resistance sources becomes essential. Some preliminary insights were provided by Fournet and colleagues, who observed that nematode populations selected on *GpaV*_*vrn*_ were not able to develop on the collinear QTL *GpaV*_*spl*_ (Fournet et al. 2013). However, the reciprocal situation remains to be evaluated. The resistance of these plants works by reducing the quality of the syncytium induced by the nematode to recover nutrients from the host plant. The consequence of restricted access to nutrients is a reduction in female production, with the population becoming predominantly male. This type of resistance is called masculinising resistance.

Such a resistance breakdown after only a few generations combined with the relatively low mutation rate in nematodes (e.g., Denver et al. 2009 for *Caenorhabditis elegans* ) suggests that the virulent alleles were already present in the initial populations used in these independent studies. Our hypothesis that virulence is the result of selection on standing variation (rather than the apparition of new mutations) is supported by this low mutation rate but also by the detection of virulent populations in different countries in Europe (Germany, The Netherlands) and by the ability of several populations (French populations [Fournet et al. 2013] but also populations from UK [Varypatakis et al. 2019, 2020]) to become virulent under independent experimental evolutions.

These facts emphasise the urgency in developing a tool to detect the presence of virulence alleles in natural populations, even at very low frequencies, and to understand the underlying mechanisms of adaptation. Both will allow to predict the adaptive potential of nematode populations and to anticipate evolutionary trajectories and contribute to limit the erosion of resistances by adapting the control strategies to any cases. However, to date only two pairs of genes, where a specific plant resistance (R) corresponded to a specific nematode effector, were identified. The first is the potato resistant gene *Gpa2* encoding for an immune receptor which recognises the *G. pallida* effector RBP‐1 (which belongs to the nematode SPRYSEC gene family) (Sacco et al. 2009) and the second is the tomato resistant gene *Cf‐2* encoding for an immune receptor which recognises the effector VAP1 from *Globodera rostochiensis* (Lozano‐Torres et al. 2012).

To address these challenges, we implemented a genome scan approach based on pooled sequencing (Pool‐Seq) to identify genomic regions under selection in *G. pallida* populations adapted to different resistance sources. Contrary to the other evolutionary forces which affect the genome in its entirety, selection is expected to leave locus‐specific signatures (Luikart et al. 2003; Storz 2005). Previous works using an Evolve and Resequence (E&R) framework on the potato cyst nematode (Eoche‐Bosy et al. 2017) and on the soybean cyst nematode (Kwon et al. 2024) have demonstrated the relevance of such methods for detecting loci under selection in response to host resistance.

With the aim of detecting and comparing genomic regions subject to selection in *G. pallida* populations adapted to the *GpaV*_*vrn*_ and *GpaV*_*spl*_ resistance QTLs, we used a genome scan on populations derived from controlled experimental evolution. Assessing whether *GpaV*_*spl*_ triggers distinct adaptive responses or shares common mechanisms with *GpaV*_*vrn*_ provides crucial pieces of information on the durability of these resistances. Moreover, we identified candidate genes with functions related to nematode virulence, some of which are known to suppress plant immunity. These candidate genes provide key insights into the molecular basis of resistance overcoming and are valuable targets for further functional validation.

## 2 Materials and Methods

### 2.1 Biology of *Globodera pallida*

*Globodera pallida* is a cyst nematode belonging to the Heteroderidae family which parasitises potatoes. It is a diploid species with an obligate sexual reproduction, producing one generation per year under European climatic conditions. The life cycle begins when second‐stage juveniles (J2) hatch from cysts in response to host plant root exudates (Devine and Jones 2000). Juveniles penetrate and migrate into the host plant roots using their buccal stylet. The parasitic interaction is established through the injection of effectors into plant cells, inducing the formation of a specialised feeding structure known as the syncytium (Eves‐van den Akker 2021). This multicellular structure allows the nematodes to extract nutrients from the host plant, promoting their growth and development. The quality of the syncytium is a key factor in sex determination: a well‐developed and functional syncytium favours the production of females, whereas a less efficient syncytium leads to male differentiation (Sobczak and Golinowski 2011). Males leave the root after their development and fertilise the females, which continue to grow until reaching full maturity. After the fertilised eggs have reached maturity, the females die and form a protective cyst containing hundreds of eggs, ensuring the parasite's long‐term persistence in the soil.

### 2.2 Experimental Evolution Design

**FIGURE 1:**
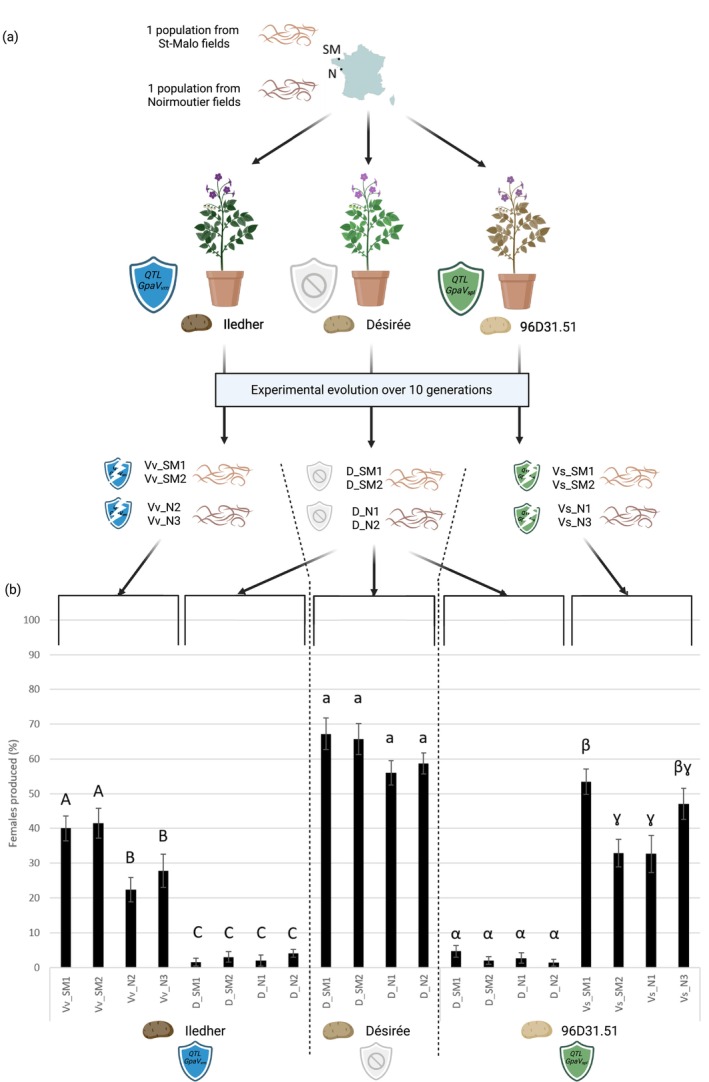
*Globodera pallida* lineages obtained by experimental evolution. (a) Two natural populations (from Saint‐Malo and Noirmoutier located in France) were developed on susceptible (Désirée) or resistant potato cultivars (Iledher containing the *GpaV*_*vrn*_ QTL and 96D31.51 containing the *GpaV*_*spl*_ QTL) during 10 successive generations. (b) The resulting *G. pallida* lineages reared on *GpaV*_*vrn*_ (Vv_SM1, Vv_SM2, Vv_N2 and Vv_N3) and lineages reared on *GpaV*_*spl*_ (Vs_SM1, Vs_SM2, Vs_N1 and Vs_N3) were inoculated on their respective resistant potato genotypes: Iledher and 96D31.51. The resulting control *G. pallida* lineages reared on Désirée (D_SM1, D_SM2, D_N1 and D_N2) were inoculated to Désirée, Ilecher and 96D31.51 to confirm the effectiveness of resistances. The virulent status of the independent lineages was confirmed on Iledher (for lineages Vv_SM1, Vv_SM2, Vv_N2 and Vv_N3) and on 96D31.51 (for lineages Vs_SM1, Vs_SM2, Vs_N1 and Vs_N3), as the lineages reared on resistances produced significantly more females than the control lineages.

The lineages were established using cysts from two natural *G. pallida* populations from infested fields near Saint‐Malo (SM) and Noirmoutier (N) in France. Each population was reared during ten successive generations on the same cultivar: the susceptible cultivar Désirée, the resistant cultivar Iledher (*GpaV*_*vrn*_) and the resistant genotype 96D31.51 (*GpaV*_*spl*_). This experiment was conducted in controlled greenhouse conditions, using the same lots of potato tubers for each host genotype to ensure uniform plant material, and consisted for each population of two or three independently evolved replicates for susceptible (Désirée) and resistant (Iledher and 96D31.51) potato cultivars, respectively. The first eight generations of experimental evolution were carried out by Lechevalier and colleagues (see Lechevalier et al. 2025 for details) in the case of lineages evolved on Désirée and Iledher. The same experimental procedure was applied in parallel to the lineages evolved on 96D31.51, ensuring equivalent conditions for all host genotypes, although they were not used in the previous study. The first generations were produced in 10‐L pots, and the pot size was gradually reduced to 1 L during the experiment. Each year, with each newly formed generation, the cysts were extracted using a Kort elutriator, ensuring the production of a single generation per year. We then extended this experimental evolution for two additional generations in 1‐L pots, thus obtaining the tenth generation for all host genotypes. Given that *G. pallida* completes one generation per year under European conditions and without overlapping generations for this species, 10 years of experimental evolution correspond to ten successive nematode generations. For each potato cultivar (Désirée, Iledher and 96D31.51) and each population of origin (SM and N), two lineages were retained for subsequent experiments (those with the highest number of cysts for the lineages produced on the resistant cultivars) and within each lineage, cysts were randomly selected for the following assays. This resulted in four control lineages evolved on Désirée (D_SM1, D_SM2, D_N1, D_N2), four lineages evolved on Iledher (Vv_SM1, Vv_SM2, Vv_N2, Vv_N3), and four lineages evolved on 96D31.51 (Vs_SM1, Vs_SM2, Vs_N1, Vs_N3) (Figure 1a).

### 2.3 Phenotyping

The *G. pallida* lineages resulting from the experimental evolution were subjected to two distinct phenotyping experiments, both following the Petri dish protocol described by Fournet and colleagues (Fournet et al. 2013). The first was designed to quantify the virulence levels of each lineage, while the second tested the hypothesis of cross‐virulence between lineages raised on Iledher (*GpaV*_*vrn*_) and 96D31.51 (*GpaV*_*spl*_). The hatching of J2 larvae was triggered by the addition of root exudate (cv. Désirée) at a minimum of 20 cysts/lineage. The cysts were placed on a 250‐μm sieve for a period of 13 days with permanent contact with the exudate. Batches of 10 newly hatched larvae were inoculated on each potato root apex with a limit of one potato per Petri dish. The number of inoculated roots ranged from 10–20 depending on the potato cultivar used. After 26 days, roots were dissected using a binocular magnifying glass to count the number of females produced.

Statistical analyses were carried out using the R software (v4.2.2). A linear model was fitted to the data to assess the effect on the number of females produced. The validity of the analyses was verified by checking the underlying assumptions of the model. The independence, normality and homogeneity of residuals were checked by Durbin–Watson, Shapiro, and Levene tests, respectively. An analysis of variance (ANOVA) was performed for each potato cultivar to assess the effect of lineages on the number of females produced. In the event of a significant difference (*p* < 0.05), a Tukey test was applied to compare means between lineages.

### 2.4 DNA Extraction and Pool‐Seq Sequencing

By pooling DNA from multiple individuals, Pool‐Seq provides highly accurate estimates of allele frequencies across the genome and is a cost‐effective alternative to individual sequencing (Futschik and Schlötterer 2010; Schlötterer et al. 2014). The optimal size of each pool was determined at 600 diploid individuals using the PIF tool, which assesses the accuracy of allele frequency estimation from pool‐based NGS population data (Gautier et al. 2013). To form pools, 200 cysts of each lineage were hydrated in sterile water and individually opened. Three J2/cyst were collected to form pools of 600 J2 for each lineage, which were stored in 1.5 mL tubes at −80°C until DNA extraction. A total of 12 pools (i.e., 3 potato cultivars [Desiree, Iledeher and 96D31.51] × 2 origins [SM and N] × 2 replicates [see Section 2.3]) were created. DNA was extracted directly from each pool using the Qiagen DNeasy Blood and Tissue Kit (Qiagen, Hilden, Germany) following the manufacturer's instructions.

Low Pass Sequencing was performed at the GeT‐PlaGe core facility (INRAE Toulouse, France). DNA quality controls were performed using the Qubit 2.0 Fluorometer and the NanoDrop 8000 Spectrophotometer, estimating an average DNA quantity of 65 ng per pool before library preparation. DNA Prep libraries were prepared according to the Illumina protocol DNA Prep, (M) Tagmentation (96 samples, IPB, 20060059). DNA was fragmented by tagmentation with bead‐linked transposomes from the library kit. Size selection was performed using Illumina Purification Beads (1/8 bead/water ratio) from the library kit. Adaptators IDT for Illumina DNA/RNA UD Indexes Set ABCD, Tagmentation (96 indexes, 96 samples respectively 20027213, 20027214, 20042666, 20042667) were ligated prior to sequencing. Library quality was assessed using a fragment analyser (Agilent) with a high sensitivity NGS kit (DNF‐474‐0500). The average library size achieved is 500 bp. DNA was sequenced on one S4 NovaSeq 6000 lane with a paired‐end read length of 2 × 150 bp using the Illumina S4 Reagent Kit v1.5 (300 cycles) (20028312). All raw sequencing files generated have been deposited in the NCBI Sequence Read Archive (PRJEB90550).

### 2.5 Read Filtering to SNP Calling

Sequencing of the 12 pools, each consisting of 600 diploid individuals, produced an average of 249 million reads/sample. Quality control and filtering of raw reads were performed using *FastQC* v0.11.7 (Andrews 2015) and *fastp* v0.20.0 (Chen, Zhou, et al. 2018) to eliminate low‐quality bases (Phred‐quality score < 30) and residual adapters. An average of 92.26% reads were correctly mapped against the *G. pallida* D383 reference genome (van Steenbrugge et al. 2023) using the *mem* algorithm from *BWA* v0.7.17 (Li 2013) with default settings. This reference genome, with a total size of 113 Mb, is assembled into 163 scaffolds (N50 = 2.9 Mb). The resulting BAM files were sorted and duplicates removed using *Picard* tool v2.18.2 (https://broadinstitute.github.io/picard). Reads not properly aligned were discarded with *Samtools* v1.6 (Li et al. 2009) and statistics associated with BAM files were calculated with *flagstat* and *coverage* commands. After filtering and removal of duplicate reads, each library contained an average of 139 million reads, representing an average depth of 184X on the D383 reference genome.

Variant calling was performed using the haplotype caller implemented in *FreeBayes* v1.1.0 (Garrison and Marth 2012) with the following options ‐K ‐C 1 ‐F 0.01 ‐G 5 ‐n 4 ‐m 30 ‐q 20. Note that some of the multi‐nucleotide polymorphisms (MNPs) generated by adjacent (bi‐allelic) SNPs that were called under these settings were further atomised using a custom *awk* script. The resulting vcf file was parsed with the *vcf2pooldata* function of the R package *poolfstat* (v2.2.0) (Gautier et al. 2022), and the corresponding *pooldata* object was constructed with options min.maf = 0.01, min.cov.per.pool = 20 and max.cov.per.pool = 400. A number of 2,378,173 SNPs were retained by *poolfstat* for the 12 lineages studied, corresponding to an average of one SNP every 48 bases across the 113 Mb reference genome.

### 2.6 Genome Scan to Identify Regions Subject to Selection

Pairwise *F*_ST_ was estimated with the *computeFST* function of *poolfstat*. As recommended in the *BayPass* manual, SNPs were subsampled using the *pooldata2genobaypass* function. Specifically, 31 subsamples of around 75,000 SNPs were generated by selecting one SNP every 31 sites along the genome. The sub‐datasets obtained were analysed in parallel with *BayPass* v2.41 (Gautier 2015) to detect genomic signatures of adaptive differentiation (based on the *X* ^*T*^ *X* statistics) and to carry out an analysis of association with the virulence/avirulence status (based on the *C*_2_ contrast statistic). To evaluate the reproducibility of the results, each analysis was independently repeated three times specifying a different seed for the Random Number Generator (−seed option). The *X* ^*T*^ *X* and *C*_2_ statistics were calculated according to the core model (CORE) and the standard covariate model (STDIS) implemented in *BayPass*. The core model (CORE), initially developed as a multivariate generalisation of the model of Nicholson et al. (2002) and Coop et al. (2010), estimates the scaled covariance matrix (Ω) of allele frequencies in populations. This model explicitly considers the covariance structure of allele frequencies in populations (through the estimation of Ω) resulting from the demographic history of populations. The *X* ^*T*^ *X* statistic estimated with the core model measures an increase in allelic frequency differentiation compared with expectations under a neutral model, while accounting for the covariance structure resulting from shared population history (Günther and Coop 2013). High values indicate loci potentially subject to selection. The standard covariate model (STDIS) runs when a covariate file is provided using the ‐efile option. This model evaluates the extent to which a covariate in a population is associated with each marker. An analysis with the *C*_2_ statistic, using the standard covariate model, was carried out to compare standardised population allele frequencies between the two population groups defined by a binary covariate determining the virulence status (−1 = avirulent, 1 = virulent) (Olazcuaga et al. 2020). This analysis identified loci for which allele frequency was significantly associated with the virulence phenotype.

To improve the detection of selection signals and the delineation of differentiated or associated genomic windows, a local score was applied to *X* ^*T*^ *X* or *C*_2_ statistics, using the method described by Fariello et al. (2017) as implemented in the compute.local.scores R function available in the *BayPass* software package (v3.0) run with default options. This approach allows us to take linkage disequilibrium into account in order to detect regions showing genetic differentiation between populations, by cumulatively integrating association signals (i.e., *p*‐values) rather than relying solely on individual markers. By applying this score, significant signals are amplified when they are consistent across several neighbouring positions, while reducing the impact of isolated false positives. This method improves the robustness of the results and facilitates the identification of regions subject to selection and has been validated by simulations with differences in drift levels, SNP density, selection intensity, and initial frequency of the selected allele (Fariello et al. 2017).

The results of three independent analyses were merged for each type of local score analysis (*X* ^*T*^ *X* or *C*_2_) to retain only the SNPs common to all three and appearing in the signals of the significant regions. Candidate regions were then selected by combining the regions identified with both local score *X* ^*T*^ *X* and local score *C*_2_.

### 2.7 Identification of Candidate Genes Involved in Adaptations

The content of genomic regions identified as subjected to selection using the local score approach was analysed in further detail. A correlation analysis was carried out between the allelic frequency of variants and the virulence levels obtained through phenotyping. For each comparison (Désirée vs. Iledher and Désirée vs. 96D31.51), the correlation between allele frequency and the average percentage of females observed on the corresponding resistant potato (Iledher or 96D31.51) was tested independently for each lineage. A linear regression model was applied to evaluate this relationship using R software (v4.2.2). SNPs with a *p* < 0.05 and an *R* ^2^ > 0.6 were retained for further analysis. This threshold ensures that correlations are significant and explain a sufficient proportion of the observed variance, thus reducing the risk of false positives. The selected SNPs were then annotated using *SnpEff* v5.2.1 (Cingolani et al. 2012) to identify the implicated genes and predict the functional impact of the variants. An analysis of secretion patterns was performed on the identified genes to detect any secreted effectors. The presence or absence of signal peptides (SP) and transmembrane domains (TM) were predicted using Phobius v1.01 (Käll et al. 2004).

## 3 Results

### 3.1 Phenotypic Adaptation to Resistances

Experimental evolution over 10 generations resulted in 12 independent *G. pallida* lineages selected from St‐Malo (SM) or Noirmoutier (N) populations. Their virulence level was measured by phenotyping them on the cultivars used to select them (Désirée, Iledher, 96D31.51). The four lineages reared on the susceptible cultivar Désirée (D_SM1, D_SM2, D_N1, D_N2) produced an average of 61.9% females on the control cultivar Désirée (Figure 1b).

Lineages reared on the resistant cultivar Iledher (Vv_SM1, Vv_SM2, Vv_N2, Vv_N3) produced significantly more females when tested on this cultivar (34% on average) compared to those reared on Désirée, which produced only 2.6% (*F*_7,145_ = 39.39 and *p* < 0.0001; Figure 1b). Similarly, lineages reared on the resistant cultivar 96D31.51 (Vs_SM1, Vs_SM2, Vs_N1, Vs_N3) produced an average of 41.5% females on this cultivar, compared to only 2.7% females produced by the lineages reared on Désirée (*F*_7,118_ = 44.85 and *p* < 0.0001; Figure 1b). These results demonstrate an adaptation to the resistances mediated by both QTLs *GpaV*_*vrn*_ and *GpaV*_*spl*_ in all independent lineages reared after 10 generations, whatever their geographical origin (SM or N).

### 3.2 Genetic Structure of Populations

The heatmap of the matrix of pairwise‐population *F*_ST_ generated from *poolfstat* (Figure S1) revealed a strong genetic differentiation between samples from the two initial populations (SM and N), with values ranging from 0.129–0.159, reflecting the geographic structuring and distinct evolutionary histories of these populations. In contrast, only a weak genetic separation was observed according to the virulence status, with values ranging from 0.016–0.046. This differentiation is weak but indicates that there was a genetic signal associated with the virulence status of lineages. As expected, independent replicates were genetically close and confirmed the repeatability of lineages reared on the same potato genotypes.

### 3.3 Genomic Regions Involved in Resistance Adaptation

**FIGURE 2:**
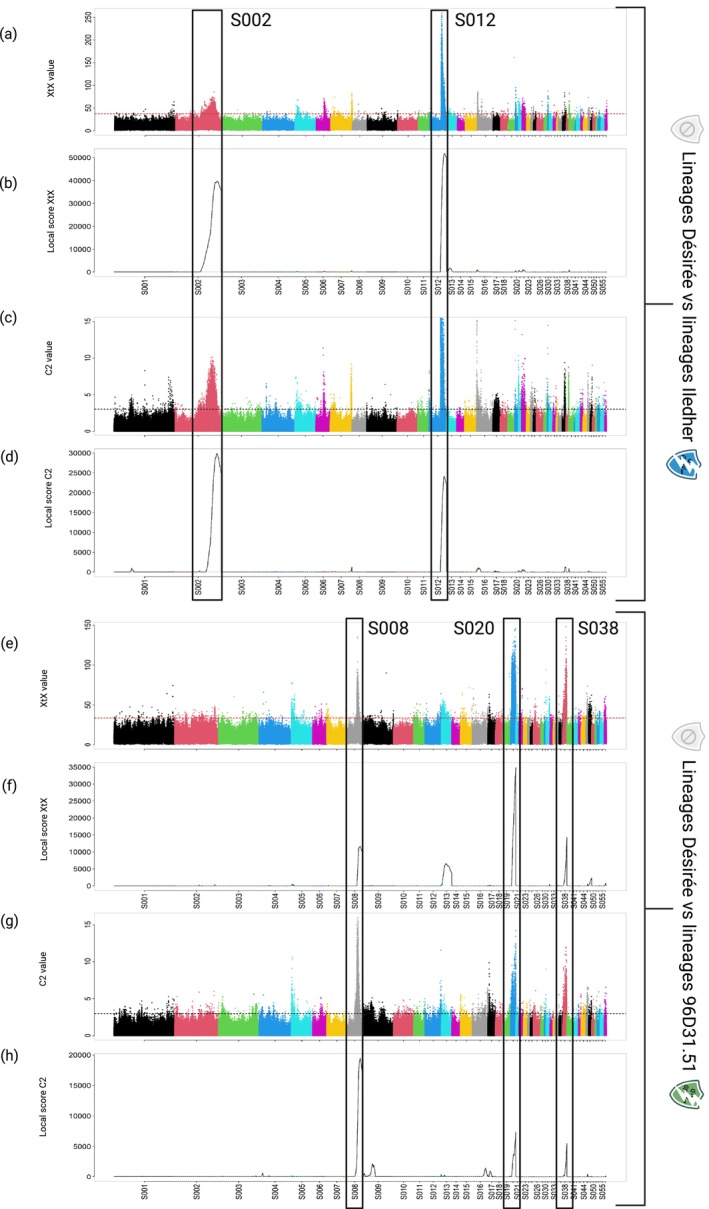
Manhattan plots showing selection signatures in the *Globodera pallida* genome detected between virulent and avirulent lineages. The Manhattan plots (a) through (d) were constructed using SNPs from the comparison between Iledher and Désirée lineages (i.e., adaptation to *GpaV*_*vrn*_), and the manhattan plots (e) through (h) from the comparison between 96D31.51 and Désirée lineages (i.e., adaptation to *GpaV*_*spl*_). Along the scaffold numbers on the horizontal axis, the vertical axis shows for each SNP the *X* ^*T*^ *X* values for (a) and (e), the *C*_2_ values for (c) and (h), the *X* ^*T*^ *X* local score values for (b) and (f) and the *C*_2_ local score values for (d) and (h). Boxes highlight scaffolds containing regions with strong evidence of selection. SNPs located above the red and black horizontal lines correspond to those exceeding the significance threshold that is, above the 95th percentile for *X* ^*T*^ *X* and beyond the 0.001 *p*‐value threshold for *C*_2_, and are therefore likely to be involved in the selection process.

Two separate analyses comparing Ildeher lineage (Vv_SM1, Vv_SM2, Vv_N2, Vv_N3) or 96D31.51 lineage (Vs_SM1, Vs_SM2, Vs_N1, Vs_N3) with control lineage Désirée (D_SM1, D_SM2, D_N1, D_N2) were performed to identify the genomic regions involved in resistance to the *GpaV*_*vrn*_ and *GpaV*_*spl*_ QTLs respectively. The genome scan analysis to identify selection signatures in genomic regions of *G. pallida* lineages was carried out using the *X* ^*T*^ *X* statistic (Figure 2a,e) and the association with the virulence/avirulence status of lineages was carried out using *C*_2_ statistic (Figure 2c,g). The convergence of the *X* ^*T*^ *X* and *C*_2_ signals reinforces the interpretation that host resistance is the main selective pressure shaping genetic differentiation in our experimental evolution.

To refine these results, a local score was applied to both statistics. On one hand, regions on scaffolds S002 and S012 were found to be the most robust candidates to assume a central role in nematode adaptation to *GpaV*_*vrn*_ resistance (Figure 2b,d). On the other hand, the regions on scaffolds S008, S020 and S038 appeared promising for studying adaptation to *GpaV*_*spl*_ resistance (Figure 2f,h). The superposition of the regions scanned obtained from all analyses, for the two comparisons (Désirée lineages vs. Iledher lineages and Désirées lineages vs. 96d31.51 lineages) reveals the absence of common genomic regions with signatures of association. This difference suggests distinct mechanisms impacting adaptation to *GpaV*_*vrn*_ and to *GpaV*_*spl*_.

### 3.4 Test for Cross Virulence

To test the existence of a cross adaptation between both resistances, a new phenotyping test for virulence was performed for both *GpaV*_*vrn*_ and *GpaV*_*spl*_ virulent lineages on respectively Ilheder (*GpaV*_*spl*_) and 96d31.51 (*GpaV*_*vrn*_) resistant cultivars.

**FIGURE 3:**
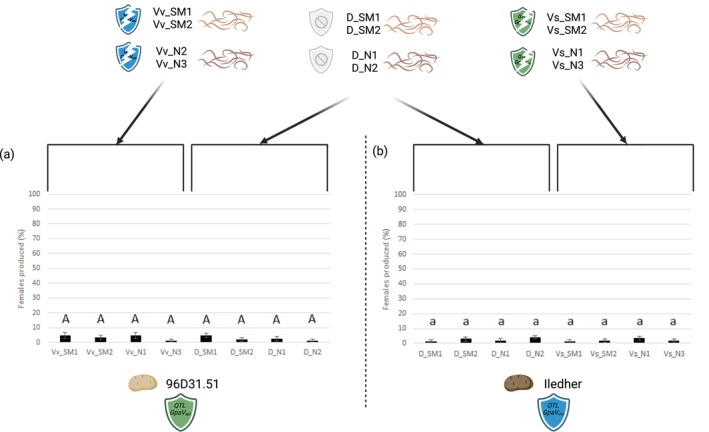
The four lineages adapted to *GpaV*_*vrn*_ (Vv_SM1, Vv_SM2, Vv_N2 and Vv_N3) were inoculated to 96D31.51 (with *GpaV*_*spl*_ QTL), and the four lineages adapted to *GpaV*_*spl*_ (Vs_SM1, Vs_SM2, Vs_N1 and Vs_N3) were inoculated to Iledher (with *GpaV*_*vrn*_ QTL). The comparison of the percentage of females produced with the four control lineages highlighted an absence of cross‐virulence in both directions.

Lineages adapted to Iledher (Vv_SM1, Vv_SM2, Vv_N2, Vv_N3) produced an average of 3.5% females on 96D31.51 (Figure 3a), while lineages adapted to 96D31.51 (Vs_SM1, Vs_SM2, Vs_N1, Vs_N3) produced 2.3% females on Iledher (Figure 3b). The control lineages reared on Désirée (D_SM1, D_SM2, D_N1, D_N2) produced only 2.7% and 2.6% respectively on these genotypes (Figure 3a,b). No significant differences in female production were observed between the different lineage groups (*F*_7,152_ = 0.58, *p* = 0.77 for 96D31.51 and *F*_7,112_ = 0.87, *p* = 0.53 for Iledher), indicating that pre‐adaptation to one resistance conferred no performance gain on the other. These results support the absence of cross‐virulence between *GpaV*_*vrn*_ and *GpaV*_*spl*_.

### 3.5 Identification of Candidate Genes for the Adaptation to *GpaV*_*vrn*_

The genome scan identified a signal of association with resistance on scaffolds S002 and S012, specific to adaptation to *GpaV*_*vrn*_. On scaffold S002, the top region is located from position 4,136,439 to 6,808,588 bp, as defined by the overlap of local score *X* ^*T*^ *X* and local score *C*_2_ results. This 2.67 Mb region contains 34,992 SNPs and encompasses 355 predicted genes. On scaffold S012, the significant region of association extends from 1,848,734 to 3,515,862 bp, corresponding to a 1.67 Mb interval with 14,053 SNPs and 188 genes.

**FIGURE 4:**
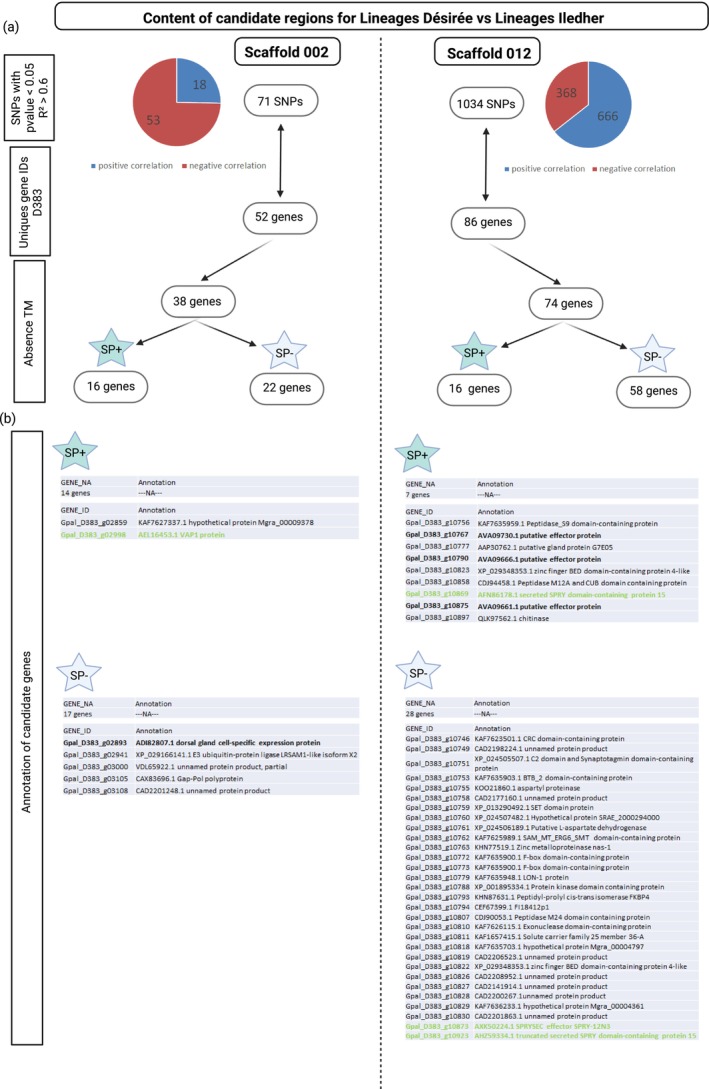
*Globodera pallida* candidate virulence genes identified on scaffolds S002 and S012 from the comparison between Iledher lineages and Désirée lineages. (a) Number of SNPs with a significant correlation and the corresponding number of genes which were filtered for the absence of transmembrane (TM) domain. (b) Genes were then put through two categories: (SP+) for the presence of a signal peptide and (SP−) for the absence of a signal peptide.

Correlation between allelic frequencies and phenotyping data enabled selection to be reduced to 71 SNPs for scaffold S002 and 1034 SNPs for scaffold S012, satisfying the condition of a *p* < 0.05 and an *R* ^2^ > 0.6 (Figure 4a and details in Table S1). Those SNPs corresponded to an association with gene IDs numbering 49 and 86 unique genes for S002 and S012, respectively. The absence of transmembrane signals specific but presence of a peptide signal, two hallmarks for plant‐parasitic nematode effectors, was verified, reducing our number of candidate genes to only 16 genes in both scaffold S002 and scaffold S012 (Figure 4b). Unfortunately, only two of the candidate genes found in scaffold S002 have a functional annotation, while nine of the candidate genes found in scaffold S012 have a functional annotation.

### 3.6 Identification of Candidate Genes for the Adaptation to *GpaV*_*spl*_

Adaptation to *GpaV*_*spl*_ by lineages reared on 96D31.51 revealed significantly associated regions on scaffolds S008, S020 and S038. The region on scaffold S008 spans from 2,291,051 to 3,470,240 bp, based on the combination of local score *X* ^*T*^ *X* and *C*_2_ analyses. This 1.18 Mb interval contains 14,387 SNPs and 242 genes. On scaffold S020, the selected region extends from 634,902 to 1,255,470 bp (620 kb), including 9819 SNPs and 112 genes. On scaffold S038, the overlapping region ranges from 521,754 to 1,036,711 bp (515 kb), with 7784 SNPs and 94 genes. These consistent results across methods highlight genomic regions potentially involved in the nematode's adaptation to the *GpaV*_*spl*_ resistance.

**FIGURE 5:**
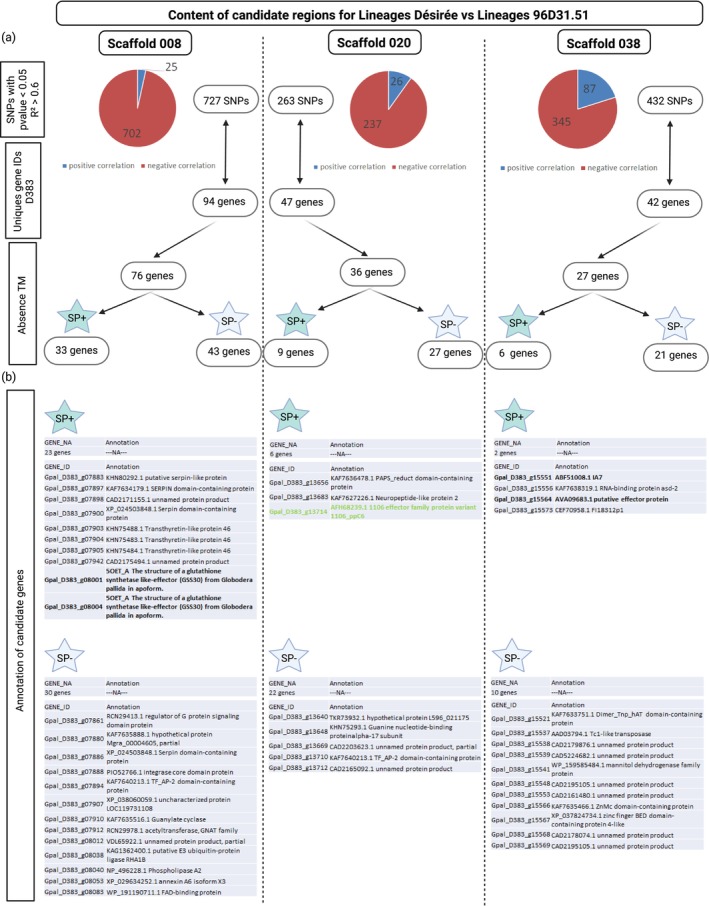
*Globodera pallida* candidate virulence genes identified on scaffolds S008, S020, and S038 from the comparison between 96D31.51 lineages and Désirée lineages. (a) Number of SNPs with a significant correlation and the corresponding number of genes which were filtered for the absence of transmembrane (TM) domain. (b) Genes were then put through two categories: (SP+) for the presence of a signal peptide and (SP−) for the absence of a signal peptide.

Among the SNPs showing a significant correlation with virulence (*p* < 0.05 and *R* ^2^ > 0.6), 727, 263 and 432 were identified in scaffolds S008, S020 and S038, respectively. Based on their predicted protein characteristics, absence of transmembrane domains and presence of a signal peptide, 33, nine, and six genes were retained in these scaffolds, suggesting their potential to be secreted and interact with the host plant (Figure 5a; details in Table S1). Among these, 10, three, and four genes from scaffolds S008, S020, and S038, respectively, were annotated (Figure 5b).

## 4 Discussion

The present population genomics study using *G. pallida* lineages derived from experimental evolution allowed us to detect genomic regions significantly associated with adaptation to two potato resistance factors, the colinear QTLs *GpaV*_*vrn*_ and *GpaV*_*spl*_. Genome scan analyses enabled us to confidently identify two genomic regions located on scaffolds S002 and S012 for the adaptation to *GpaV*_*vrn*_, and three genomic regions located on scaffolds S008, S020 and S038 for the adaptation to *GpaV*_*spl*_. The absence of genomic regions in common between the two adaptations led us to predict and to test the absence of cross virulence. Our phenotyping results confirmed that adaptation to one resistance did not confer an adaptive advantage to the other. Finally, our stepwise filtering strategy allowed us to identify candidate genes, and in particular potential effectors coding for proteins secreted by nematodes to interact with their host (e.g., Mitchum et al. 2013).

### 4.1 Testing the Reliability of the Analyses of Association

To perform a genome scan and detect regions associated with virulence, we used two complementary statistics, *X* ^*T*^ *X* and *C*_2_, both implemented in *BayPass*. The *X* ^*T*^ *X* statistic was used to detect selection signatures without a priori. The *C*_2_ statistic was then used to validate that the genetic differentiation observed corresponded to selection exerted by resistances during the experimental evolution. Since both methods rely on differences in allele frequency between the same comparison groups, an overlap in signals was not surprising. However, the combined use of these two statistics allowed us to confirm that the differentiation observed was indeed consistent with selection induced by the resistant QTLs and not differentiation linked to other sources of uncontrolled selection (e.g., co‐infection with another pathogen).

To test the accuracy of our analyses, we repeated the analyses after swapping the identities of the lineages in the *C*_2_ parameters that is, artificially treating some avirulent lineages as virulent and vice versa. For the comparison between Iledher and Désirée, the virulence status was swapped between D_SM1 and Vv_SM1, and between D_N1 and Vv_N2. For the comparison between 96D31.51 and Désirée, virulence status was swapped between D_SM1 and Vs_SM1, as well as between D_N1 and Vs_N1. In these permuted datasets, the *C*_2_ statistic and the associated local score revealed none of the previously identified traces of selection (Figure S2), confirming the robustness of our analyses.

The *F*_*ST*_ were calculated independently within SM or N lineages, to verify that the association signals observed were not simply due to the origin of the population (Figure S3). As the profile of the outlier regions was similar within each population and using the association analysis which considers the genetic structure of populations, the hypothesis that adaptation is due to the selection of standing variation(s), rather than the apparition of novel mutation(s) seems to be confirmed. Indeed, the probability of finding the two same genomic regions (for the adaptation to *GpaV*_*vrn*_) or the three same genomic regions (for the adaptation to *GpaV*_*spl*_) in both SM and N lineages by chance only is extremely low. This result also illustrates the necessity of using a tool like *BayPass* that considers the genetic structure of populations, a crucial step considering the geographical divergence of the studied populations (Gautier 2015). While our results highlight robust patterns across the sampled populations, it remains to be tested whether similar regions would be involved in lineages originating from other parts of the world, such as South America, where *G. pallida* originally comes from (Plantard et al. 2008).

### 4.2 Limitations Due to Short‐Read Sequencing and to Quality of the Reference Genome

Our analyses are limited by the use of short‐read sequencing data, which prevents the detection of structural variants such as insertions, deletions, or copy number variations, which could also be highly relevant in effector diversification. Such structural changes have already been shown to play a key role in virulence evolution. For instance, in *Leptosphaeria maculans*, a deletion affecting a genomic region led to the complete loss of an avirulence gene, thereby allowing the pathogen to evade host recognition and become virulent (Balesdent et al. 2013). More broadly, multiple molecular pathways can lead to the breakdown of plant resistance, with virulence arising via distinct genetic mechanisms within pathogen populations (Rouxel and Balesdent 2017). These examples underscore the necessity of adopting long‐read sequencing approaches to better characterise structural variations, particularly copy number variations (CNVs), which may play a key role in effector diversification and virulence evolution in nematodes (Castagnone‐Sereno et al. 2019).

Moreover, as the D383 reference genome is at the scale of scaffolds (van Steenbrugge et al. 2023), the actual chromosomal position of these regions remains uncertain. Peaks highlighted in different scaffolds could belong to the same chromosome, raising the question of whether the observed adaptations are due to a monogenic or to a polygenic architecture. Further assembly at chromosomal level would be required to clarify this point.

Another significant limitation of this reference genome is the high proportion of candidate genes lacking functional annotation (NA). This limits the biological insights that can be extracted from certain genomic regions. Notably, in our dataset, SNPs with the strongest *C*_2_ values (i.e., the most significant associations with virulence) corresponded to NAs. Although the additional filter we applied may have excluded some of these potentially relevant candidates, it enabled us to focus on a shortlist of genes with possible functional interpretation.

### 4.3 Absence of Cross‐Virulence Between Resistances: Implications for Predictive Resistance Deployment Strategies

While both *GpaV*_*vrn*_ and *GpaV*_*spl*_ conferred strong resistance to the initial avirulent lineage, virulent phenotypes emerged after ten generations of experimental evolution. Genome scans revealed distinct selection signatures for adaptation to *GpaV*_*vrn*_ and *GpaV*_*spl*_, showing that resistance breakdown occurred differently for each case. Importantly, no cross‐virulence was detected between the two resistances, indicating that adaptation to one resistance does not confer any adaptive advantage for overcoming the other. This result confirms previous findings by Fournet and colleagues, who showed that a *G. pallida* lineage selected on *GpaV*_*vrn*_ was unable to produce females on potato genotypes carrying *GpaV*_*spl*_ (Fournet et al. 2013). Our reciprocal approach now demonstrates that lineages selected on *GpaV*_*spl*_ are also unable to overcome *GpaV*_*vrn*_, and those results were clearly explained by the fact that the genomic regions involved in adaptation to each resistance were distinct. Although the selected regions differ, the selection process probably follows a similar mechanism: virulent individuals are present at low frequencies in the population and are gradually selected under the pressure of resistance. This process needs several generations because the fact that avirulent individuals can develop into males may slow the spread of virulence by maintaining avirulent alleles in the population (Tankam Chedjou et al. 2024). The colinear position of *GpaV*_*vrn*_ and *GpaV*_*spl*_ on the potato V chromosome was not sufficient to induce cross‐virulence between the two resistances, suggesting that physical proximity alone is not sufficient to observe cross‐virulence. Altogether, the concordance between cross‐virulence results and the detection of selection signals obtained with genome scans illustrates the interest of predictive genomics to anticipate virulence evolution and supports its application in resistance management strategies.

The specificity of the genomic regions involved in adaptation to each source of resistance offers the possibility of deploying *GpaV*_*spl*_ in populations already virulent to *GpaV*_*vrn*_, and vice versa. This last scenario remains hypothetical for the moment, as *GpaV*_*spl*_ is not yet widely deployed in the field and because no qualitative epidemiological surveillance has been conducted to assess the virulence status of natural populations on this resistance source. Our experimental evolution shows that *GpaV*_*spl*_ can be overcome within ten generations, in the same way as for *GpaV*_*vrn*_. This rapid breakdown suggests that *GpaV*_*spl*_ is not more durable than *GpaV*_*vrn*_, but simply benefits from its limited use today. Consequently, in the absence of protective measures, the erosion observed for *GpaV*_*vrn*_ could also occur for *GpaV*_*spl*_ if deployed alone. A promising approach to improve the durability of *GpaV*_*spl*_ is its combination with other factors of resistance, such as the quantitative QTL *GpaXI*_*spl*_ and the combination of both *GpaV*_*spl*_ and *GpaXI*_*spl*_; an association which was shown to be more resistant (Caromel et al. 2005; Rouppe van der Voort et al. 2000).

The effectiveness of pyramiding strategies in increasing the effectiveness and durability of resistance has been well documented in various pathosystems (see Pilet‐Nayel et al. 2017 for a review). For instance, in pepper, combining a major resistance gene with quantitative resistance loci enhanced both resistance levels and their long‐term durability (Quenouille et al. 2013). Studies in apple scab have demonstrated that pyramiding multiple quantitative resistance loci significantly improves resistance efficiency against apple scab and may contribute to enhanced durability (Laloi et al. 2017).

These results, observations and future prospects underline the importance of developing molecular predictive tools able to track the frequency of virulence alleles to anticipate the adaptive potential of nematode populations and to design deployment strategies based on the virulence profiles of local populations. The implementation of such tools could guide breeders in selecting the most appropriate and durable resistance combinations to include in their potato cultivars. But their most immediate value could be in helping farmers to make the right decisions. By guiding the choice of resistant varieties best suited to a region's virulence context, these tools would enable more sustainable deployment, with the aim of delaying the emergence of overcome and improving long‐term control of *G. pallida*.

### 4.4 Effector Candidates Involved in Adaptation to *GpaV*_*vrn*_ and *GpaV*_*spl*_ Resistances

Regarding the candidate genes identified in the genomic regions associated with adaptation to *GpaV*_*vrn*_ resistance, some of them had already been identified as effectors in previous studies. Among them, our results revealed on scaffold S002 a candidate effector known to be potentially involved in suppressing plant immunity and named as venom allergen‐like protein (VAP1). The SNP linked to this gene shows a negative correlation slope, indicating that the reference allele is less frequent in virulent lineages, consistent with its replacement under selection pressure and supporting a possible contribution to virulence. In *G. rostochiensis*, VAP1 has been shown to interfere with immune signalling initiated by pattern recognition receptors on the surface of plant cells, attenuating early defence responses (Li et al. 2021; Lozano‐Torres et al. 2014). Their ability to suppress the PTI response, the immune response triggered by a molecular motif associated with pathogens, has also been confirmed by functional screens (Pogorelko et al. 2020).

Three effectors located in S012 were previously characterised as putative effectors in *Heterodera avenae* (Chen, Chen, et al. 2018). These genes were shown to encode secreted proteins able to modulate cell death in *Nicotiana benthamiana*, suggesting a potential role in host–pathogen interactions. Another more relevant gene, identified on the S012 scaffold, encodes a secreted protein containing a SPRY domain, which is a member of the SPRYSEC effector family. The SPRYSEC identified harbours a SNP whose allele frequency is negatively correlated with virulence levels. The SNP in this gene shows a negative correlation slope, indicating that the reference allele is less frequent in virulent lineages, consistent with its replacement under selection pressure and suggesting a possible contribution to virulence. The SPRY domain functions as a protein‐binding module, enabling these effectors to interact with various host proteins and disrupt defence‐related complexes (Diaz‐Granados et al. 2016). SPRYSEC proteins are known to be involved in suppressing cell death and thus inhibiting ETI‐related plant defence responses (Ali et al. 2015; Mei et al. 2015; Postma et al. 2012; Sacco et al. 2009). The SPRY domain‐containing secreted protein 15 detected in this study is able to bind and inhibit some NLRs (nucleotide binding and leucine‐rich repeat), preventing immune signalling from the host plant (Ali et al. 2015; Contreras et al. 2023; Sugihara et al. 2025).

The presence of VAP1 and SPRYSEC was also observed in a previous transcriptomics study comparing the same avirulent lineages reared on Désirée with virulent lineages reared on *GpaV*_*vrn*_ (Lechevalier et al. 2025). Their consistent detection across both genomic and transcriptomic studies strongly supports their role in overcoming the resistance mechanisms conferred by *GpaV*_*vrn*_. On the other hand, unannotated genes should not be overlooked, as several of them were also detected in the transcriptomic study. For example, the Gpal_D383_g02789 gene located on the S002 scaffold, which contains 21 SNPs subject to selection pressure, was found in both datasets, even though none of its SNPs passed the genomic filters. On the S012 scaffold, 90 SNPs passed the filtering steps, but they were associated with genes predicted as non‐secreted (SP−). Interestingly, these SNPs were grouped into six unannotated genes (Gpal_D383_g10745, g10764, g10769, g10792, g10795 and g10798), all of which were identified as differentially expressed in the transcriptomic analysis. This overlap highlights the potential biological significance of these uncharacterized genes and underscores the need for further studies to better characterise unannotated genes.

Among the candidate genes identified in genomic regions associated with adaptation to *GpaV*_*spl*_, several showed features compatible with effector proteins. In S008, two annotated genes encode known effectors. Both correspond to a glutathione synthetase‐like effector (GSS30). GS‐like effectors result from the neofunctionalisation of a housekeeping gene and are expressed in the nematode's dorsal gland and then secreted into the syncytium to support parasitism (Lilley et al. 2018).

On the S020 scaffold, a relevant gene was annotated as a member of the 1106 effector family. This cyst nematode‐specific family, also known as GLAND4, has been described as a suppressor of plant immunity and is able to bind DNA (Barnes et al. 2018; Noon et al. 2015).

Regarding genes in the S038, two were classified as effectors. The first gene annotated IA7 effector, identified in *G. pallida* , is specific to potato cyst nematodes and is secreted in subventral glands and expressed at J2 stages (Blanchard et al. 2007; Grenier et al. 2002). The GpIA7 effector of *G. pallida* reduces the expression of key cell cycle regulators in host plants and stimulates the endocycle when syncytium formation is triggered (Coke et al. 2021). The second one encodes a putative effector protein (AVA09683.1) previously identified in *Heterodera avenae* through functional screening in *Nicotiana benthamiana* (Chen, Chen, et al. 2018).

Reassuringly, some of the effectors detected in this study and potentially involved in adaptation to *GpaV*_*vrn*_ or *GpaV*_*spl*_ have also been identified in similar genome scan studies. Effectors involved in the suppression of plant immunity, such as VAP or SPRYSEC types, have likewise been detected in cases of nematode adaptation to resistance genes (Eoche‐Bosy et al. 2017; Kwon et al. 2024). Altogether, the candidate genes for the adaptation to *GpaV*_*vrn*_ and to *GpaV*_*spl*_ correspond to several well‐characterised effectors, but also to numerous genes with unknown or poorly defined functions. These results suggest that adaptation to resistance may rely on effectors that modulate plant immunity, but also on other less characterised molecular processes. While these effector families remain interesting targets for future functional validation, it will also be interesting to expand the study of unannotated genes in order to fully understand the diversity of adaptive strategies in *G. pallida* .

## Author Contributions

J.M., S.F. and E.G. conceived the study. J.M. and S.F. performed the experimental evolution. O.L. and S.F. performed the phenotyping experiments. O.L. and M.E. performed the preparation of pools and DNA extractions. O.L. and R.F. performed the libraries and the sequencing. O.L., M.E. and M.G. performed the genome scan analyses. O.L. and J.M. performed the statistical analysis of phenotypic data. All the authors have made substantial contributions to the interpretation of data. O.L. wrote the text which was edited by all the authors. All authors have approved the current version.

## Conflicts of Interest

The authors declare no conflicts of interest.

## Supporting information


**Data S1:** mec70105‐sup‐0001‐DataS1.zip.

## Data Availability

Raw reads of the 12 *G. pallida* lineages are available at the European Nucleotide Archive database system under the project accession number PRJEB90550 (https://www.ebi.ac.uk/ena/browser/view/PRJEB90550). The raw phenotyping results are available in the Supporting Information (Table S2). The R and bash scripts used for the analyses are also provided in the Supporting Information (Script S1–S4).
