# Supplementary material for: Adaptation of the Cyst Nematode Globodera pallida to the Colinear Potato Resistant QTLs GpaV vrn and GpaV spl Involved Distinct Genomic Regions and Absence of Cross‐Virulence
Source: Mol Ecol. 2025 Sep 13;34(21):e70105. doi: 10.1111/mec.70105 (PMC12573733; doi:10.1111/mec.70105)
Supplement: Supplementary file 1 — Data S1: mec70105‐sup‐0001‐DataS1.zip. [file MEC-34-e70105-s001.zip › supinfo/mec70105-sup-0001-TableS1-S2-FigureS1-S3@Revised_SupportingInformation_Lechevalier-et-al_MolEcol_CleanCopy.docx]

**Adaptation of the cyst nematode *Globodera pallida*** **to the colinear potato resistant QTLs *GpaV_vrn_* and *GpaV_spl_* involved distinct genomic regions and absence of cross-virulence**

Océane LECHEVALIER^1^, Magali ESQUIBET^1^, Mathieu GAUTIER^2^, Rachel FOURDIN^3^, Eric GRENIER^1^, Sylvain FOURNET^1$^ and Josselin MONTARRY^1$*^

^1^ IGEPP, INRAE, Institut Agro, Univ Rennes, Le Rheu, France

^2^ CBGP, Univ Montpellier, CIRAD, INRAE, IRD, Institut Agro Montpellier, Montpellier, France

^3^ I2MC, UMR1297, INSERM, Toulouse, France

^$^ These authors contributed equally to this work

^*^ Corresponding author: josselin.montarry@inrae.fr

**Supporting Information**

**Tab. S1** Selection of candidate SNPs contained in regions showing signs of selection and identified by local scores (S002, S012, S008, S020, S038). The table gives for each SNP, the chromosome on which it is located (CHROM), its position in Mb on the chromosome (POS), the reference allele (REF), the alternative allele (ALT), its functional impact (EFFECT), the associated gene (GENES_ID) and its annotation (ANNOTATION), the presence/absence of transmembrane signal (TM) and peptide signal (SP) for the gene concerned. The allelic frequency in each lineage (ALL_FREQ_X) is indicated. SNPs were filtered on an R² > 0.6 (R_squared) and a pvalue < 0.05 (P_VALUE), the values of which are shown with the associated slope (SLOPE).

**Tab. S2** Results of phenotyping *G. pallida* lineages on potato roots. Each row corresponds to an inoculated root. The table shows for each row the name of the lineage tested (lineage), the identifier of the inoculated root (root), and the number of females counted on this root after development (female). For each root, ten J2 larvae were inoculated at the start of the experiment.

**Script. S1** This script, run on R, provides an example of the analyses performed on phenotyping data. It includes basic visualization of the number of females per inoculated root and statistical tests to assess differences between *G. pallida* lineages.

**Script. S2** This script, run on R, provides an example of the analyses performed with the *Poolfstat* package. It includes importing VCF data, creating a *pooldata* object, exploratory analyses of the dataset, and extracting a subset of pools for *Baypass* analyses.

**Script. S3** This bash script provides an example of the commands used to perform CORE and STDIS analyses on genotype files with *Baypass*.

**Script. S4** This script, run on R, provides an example of the analyses performed on *Baypass* output files. It concatenates the results, explores the data using *X^T^X*, C_2_, BF, and local score.

**Fig. S1** Heatmap clustering of samples based on Pairwise *F*_ST_ estimated with the *computeFST* function of *poolfstat*. The heatmap includes control *G. pallida* lineages reared on Désirée (D_SM1, D_SM2, D_N1 and D_N2), lineages reared on *GpaV_vrn_* (Vv_SM1, Vv_SM2, Vv_N2 and Vv_N3) and lineages reared on *GpaV_spl_* (Vs_SM1, Vs_SM2, Vs_N1 and Vs_N3). The geographical origin of the lineage was Saint-Malo (SM) or Noirmoutier (N).

**Fig. S2** Manhattan plots showing the results of simulated analyses of virulence status permutations between different lineages within the parameters of C_2_. For the concerned lineages, the avirulent status was permuted with the virulent status and inversely. For the comparison between Iledher and Désirée (*i.e.*, adaptation to *GpaV_vrn_*), virulence status was permuted between Vv_SM1 and D_SM1, and between Vv_N2 and D_N1. For the comparison between 96D31.51 and Désirée (*i.e.*, adaptation to *GpaV_spl_*), the swap was performed between Vs_SM1 and D_SM1, and between Vs_N1 and D_N. Those permuted datasets illustrated the robustness of the analyses as peaks detected in the real datasets were not detected here.

**Fig. S3** Manhattan plots showing genome-wide pairwise *F*_ST_ values between virulent and avirulent *Globodera pallida* lineages. Plots (a) through (c) correspond to comparisons between Désirée and Iledher lineages (*i.e.* adaptation to *GpaV_vrn_*), while plots (d) through (f) correspond to comparisons between Désirée and 96D31.51 lineages (*i.e.* adaptation to *GpaV_spl_*). Analyses were performed separately for lineages derived from different initial populations: (a) and (d) include all lineages from both Saint-Malo (SM) and Noirmoutier (N), (b) and (e) include only lineages from Saint-Malo (SM), and (c) and (f) include only lineages from Noirmoutier (N). Boxes highlight scaffolds containing regions with strong evidence of selection.

**Fig. S1 – Lechevalier et al**


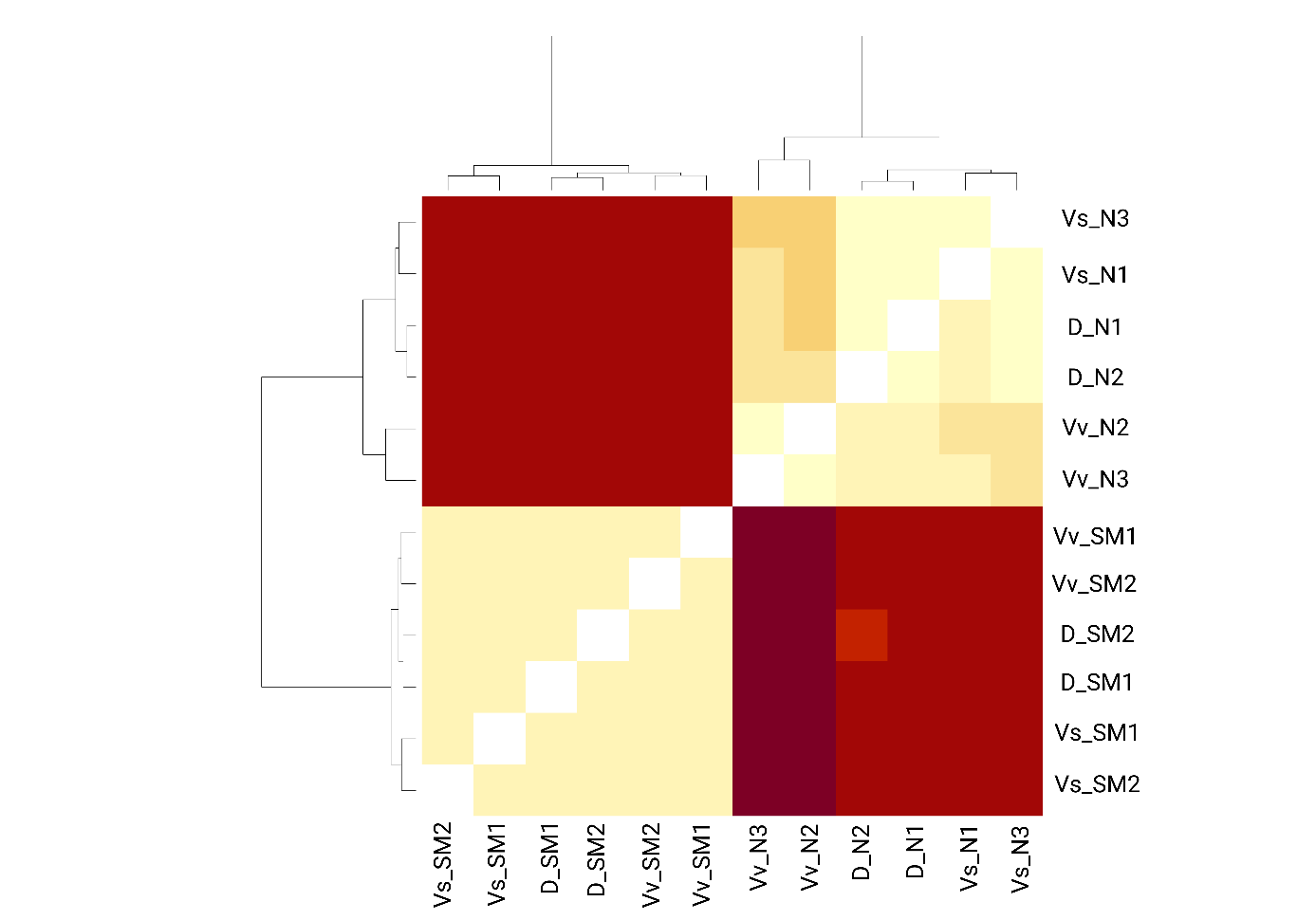


**
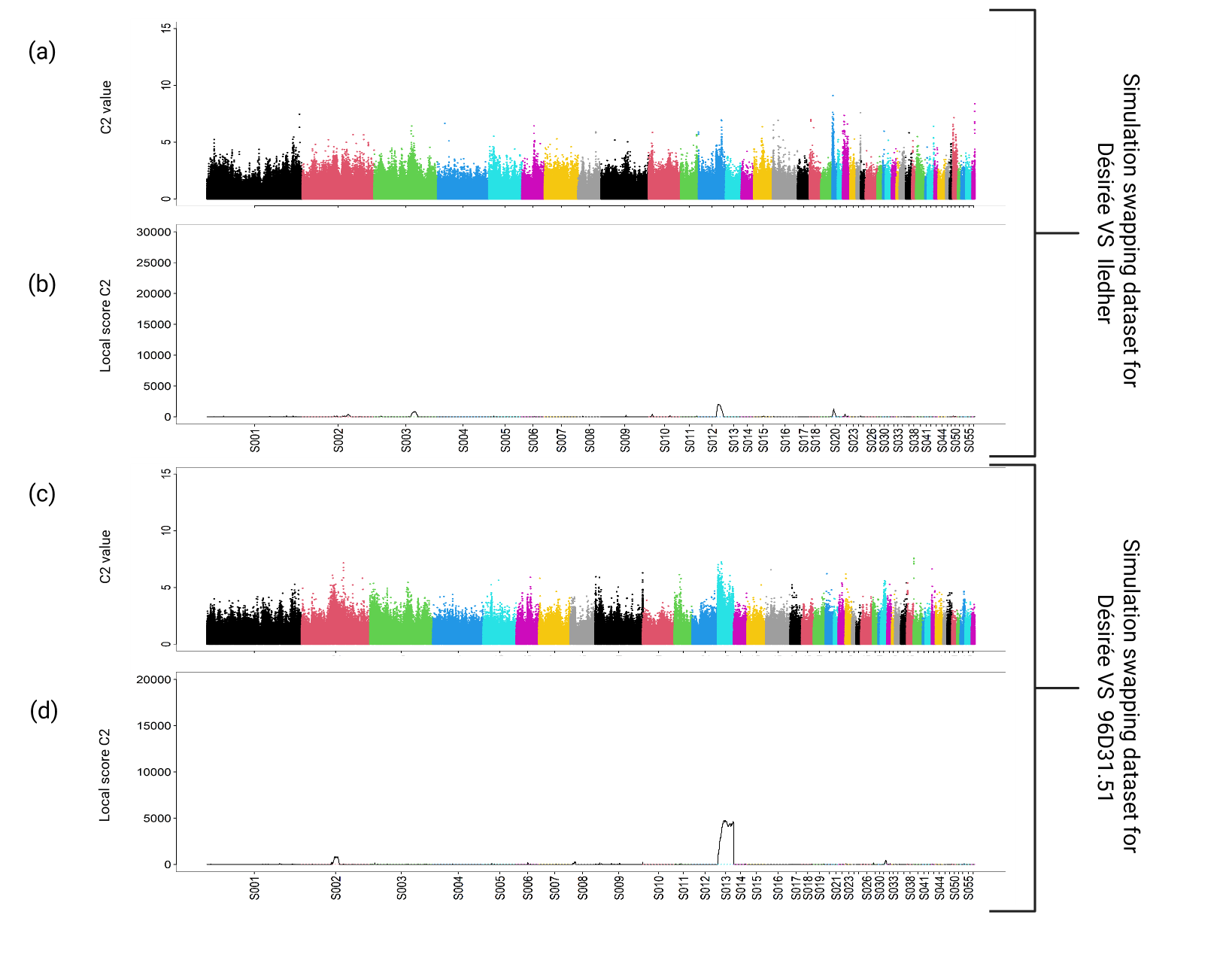
Fig. S2 – Lechevalier et al**


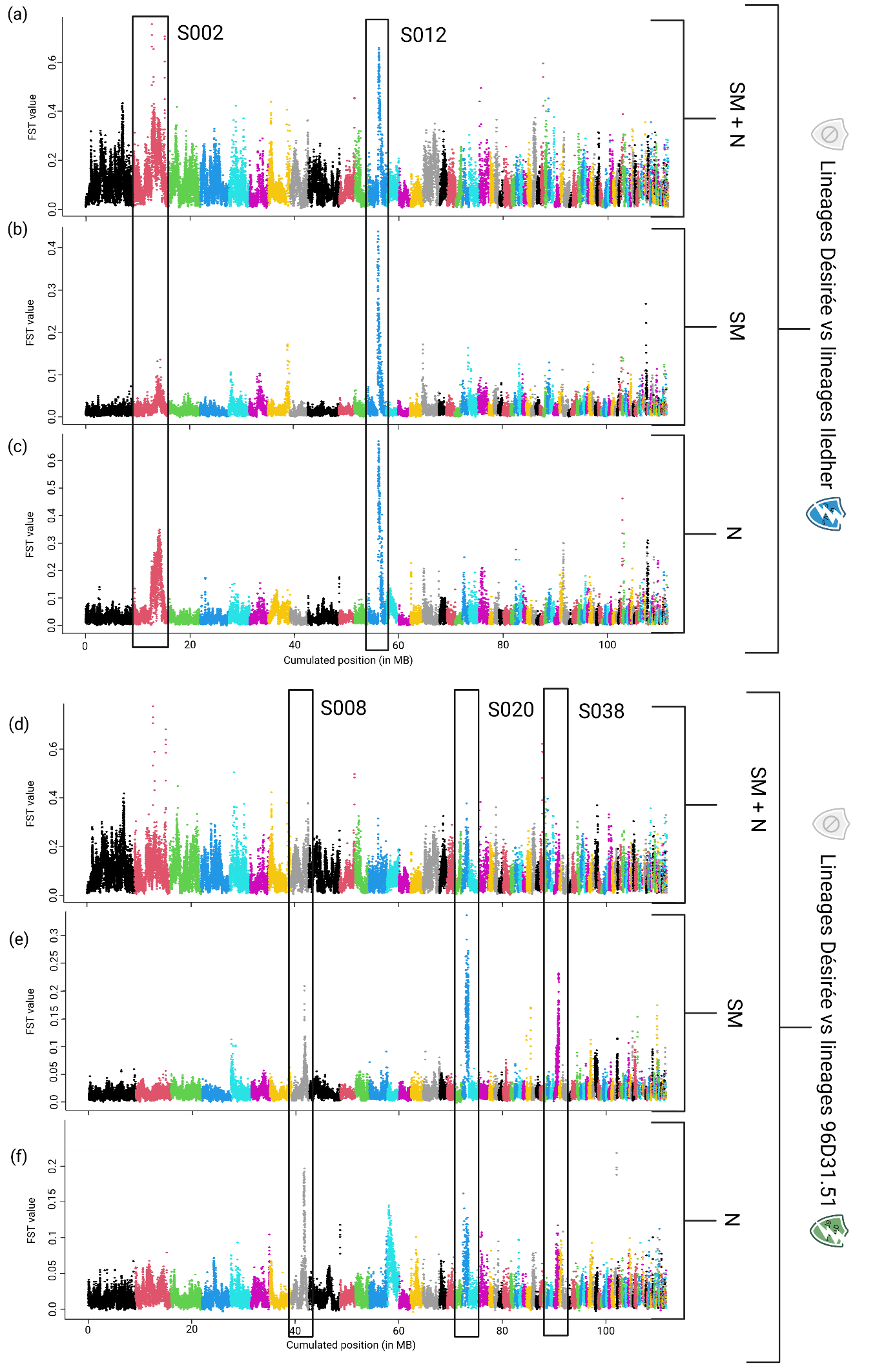
**Fig. S3 – Lechevalier et al**
